# Spectroscopic Characterization of the Photolysis of Riboflavin (Vitamin B2) via Time-Resolved Mass Spectrometry and IRMPD Spectroscopy

**DOI:** 10.1021/acs.jpca.5c02175

**Published:** 2025-05-29

**Authors:** Sarah A. Wilson, Aljawharah Alsalem, Giel Berden, Jos Oomens, Caroline E. H. Dessent

**Affiliations:** † Department of Chemistry, 8748 University of York, York YO10 5DD, U.K.; ‡ FELIX Laboratory, Institute for Molecules and Materials, 6029 Radboud University, Toernooiveld 7, Nijmegen 6525 ED, The Netherlands

## Abstract

Riboflavin, Vitamin B2, is a key photoactive biomolecule that has important uses as a food additive and as a photocatalyst. While riboflavin’s photodegradation pathways have been studied extensively, open questions exist about the effect of the chemical environment on riboflavin photodegradation and the nature of the subsequent photoproducts. Here, we use time-resolved mass spectrometry (TRMS) and gas-phase infrared multiple-photon dissociation (IRMPD) spectroscopy to characterize 365 nm online photolysis of riboflavin under basic conditions. TRMS allowed for monitoring of the light-induced decay of deprotonated riboflavin along with the formation of photoproducts and photolysis intermediates. IRMPD spectroscopy was performed over the fingerprint region (1100−1800 cm*^–^* ^1^) at the FELIX free-electron laser facility, to obtain the first gas-phase IR spectrum of deprotonated riboflavin, the isolated chromophore, along with the IRMPD spectrum of the deprotonated riboflavin dimer. In addition, spectroscopic characterization was performed for the photoproducts lumichrome and lumiflavin, as well as the photolysis intermediates formylmethylflavin and the riboflavin-lumichrome dimer. Our experiments reveal that 365 nm photolysis of the riboflavin dimer is enhanced compared with the monomer, potentially due to spectral shifting of the chromophore upon complexation. The clear propensity for formation of the dimer that we observe for riboflavin and its photolysis behavior indicates that aggregates play a significant role in accelerating photodegradation of riboflavin. This is the first time, to our knowledge, that such an effect has been identified in flavin photochemistry and provides new insight into why photodegradation of riboflavin is particularly sensitive to solution conditions.

## Graphical abstract

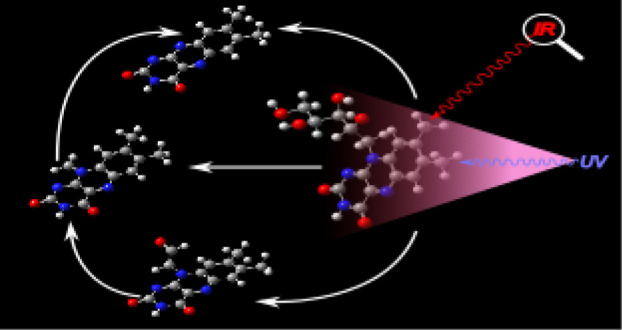

## 1 Introduction

Riboflavin (Vitamin B2) is a key member of the flavin family of biomolecules, which are renowned for their role as the light-sensitive components in photoreceptors.^1,2^ It is found in a wide variety of natural and fortified food products, including milk and cereals, and its photolytic behavior has been extensively studied due to its propensity to rapidly degrade upon light exposure. Riboflavin’s photochemical properties are also important in advanced synthetic protocols, where it has been increasingly exploited as a powerful, metal-free photocatalyst.^3^ Given the broad importance of riboflavin’s photolytic behavior, it has been subject to numerous experimental and theoretical investigations,^1,4^ with studies revealing that its photoproducts are dependent on both the chemical environment (pH, solvent, etc.) and the photolysis wavelength.^1,5,6^ Significant uncertainty remains, however, about the identity of the photoproducts and the pathways by which they are formed. Indeed, even when riboflavin’s photoproducts have been identified via their *m*/*z* in mass spectrometric analysis,^4,7,8^ questions remain about the exact molecular structure given the potential for tautomerization in these systems. Scheme 1 illustrates the geometric structure of riboflavin, along with its primary photoproducts, lumiflavin, and lumichrome.

**Scheme 1:**
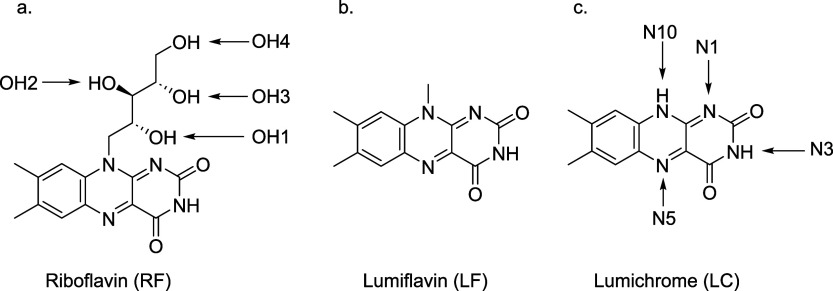
Schematic Diagram of a) Riboflavin (RF), b) Lumiflavin (LF), and c) Lumichrome (LC)^a^

a Labels are included on the riboflavin structure to identify the ribityl OH groups, and on the lumichrome structure to identify the N atoms of the flavin heterocycle.

Gas-phase IR action spectroscopy coupled to mass spectrometry is becoming an increasingly important method for structural determination of unknown molecular species involved in chemical reactions.^9–11^ Mass spectrometry provides the initial screening via *m*/*z* selection and ion isolation, and IR spectroscopy is then applied over the fingerprint region to determine the chemical structure. IR action spectra can be acquired either via IRMPD or via the messenger tagging approach.^9–11^

In this study, we use time-resolved mass spectrometry,^12,13^ coupled to infrared multiple-photon dissociation (IRMPD), to spectroscopically characterize deprotonated riboflavin and its photoproducts formed upon solution-phase photolysis at 365 nm. IRMPD spectroscopy was performed over the fingerprint region (700–1800 cm*^–^* ^1^) using the Free Electron Laser for Infrared eXperiments (FELIX), with quantum-chemical calculations conducted to aid in the assignment of geometric structures. The investigation conducted here builds on earlier work from our group, where we performed online photolysis (365 nm) measurements of riboflavin solutions, along with *in vacuo* laser photodissociation of deprotonated riboflavin.^4^ Lumiflavin and lumichrome were identified as the major photoproducts based on their *m*/*z* values, for both solution- and gas-phase photolysis, along with other minor photofragments. The IRMPD spectroscopy to be performed in the current work will allow us to definitely identify whether lumichrome and lumiflavin are the correct identities of these major photoproducts (rather than isomeric species with identical *m*/*z* values) and, importantly, also reveal the identity of lower-intensity photoproducts.

Previous gas-phase IR spectroscopy has been performed for flavin systems on protonated and metalated analogues of lumiflavin, lumichrome, and riboflavin.^14–16^ For the protonated species, the spectra show the presence of protomers, revealing the propensity of these systems for tautomerization. While these cationic flavin chromophore systems have already been studied via gas-phase IR spectroscopy, the work presented here is the first to characterize negatively charged (deprotonated) systems and will thus provide new insight into how charge variation affects the flavins’ geometric structures. We further note that there have been a number of studies of other flavin chromophores over the past decade where gas-phase electronic spectroscopy has been employed to probe the intrinsic electronic structure.^15,17–22^

## 2 Methods

Time-resolved solution-phase UV photodissociation experiments were conducted in an AmaZon SL dual funnel electrospray ionization quadrupole ion trap (ESI-QIT) mass spectrometer (Bruker Daltonics Inc., Billerica, MA, USA). Solutions of riboflavin (10*^–^* ^6^ mol*/*dm^3^) in water and 2 μL of NH_3_ were illuminated in a borosilicate glass syringe with a UV light-emitting diode (LED) of wavelength 365 nm and power between 0.88 and 1.29 W (Thorlabs M365L3), electrosprayed using typical instrumental parameters (nebulizing gas pressure of 7.0 psi; injection rate of 0.18 mL/h; drying gas flow rate of 8.0 L/min), and run in negative ion mode at a capillary temperature of 180 °C. Riboflavin was purchased from Sigma-Aldrich, and the ammonia solution used was 25%, LiChropur for HPLC, Merk Scientific.

IRMPD spectroscopy experiments were performed at the FELIX free-electron laser facility, using a modified commercial quadrupole ion-trap mass spectrometer (Bruker, AmaZon Speed ETD).^23^ Solutions of riboflavin (10*^–^* ^6^ mol*/*dm^3^) in water and 2 μL NH_3_ were introduced at 180 μL*/*h flow rates. Ions of interest were generated via ESI and mass-selected using MS/MS and fragmented by IRMPD using a single FELIX macropulse. Spectra were recorded over the 1100−1800 cm*^–^* ^1^ region, with 8–10 μs macropulses of 40–120 mJ per pulse at a 10 Hz repetition rate and with a bandwidth of 0.4% of the center frequency. Resonant absorption of IR radiation leads to an increase in the internal energy of an ion mediated by intramolecular vibrational redistribution (IVR), which eventually leads to unimolecular dissociation. After irradiation, a mass spectrum of the resulting ions in the trap is recorded. At each IR frequency point, five mass spectra were averaged. The dissociation was calculated from the mass spectra by relating the precursor ion and fragment ion intensities (eq 1) and plotted as a function of IR frequency.

1 $$IRMPD=-\ln\frac{I_{p}}{I_{p}+\sum I_{f}}$$

where *I*_p_ is the intensity of the precursor ion, and *I*_f_ is the intensity of the fragmented ions. The IRMPD intensity was linearly corrected for the frequency-dependent laser pulse energy. Spectra were recorded at two levels of laser-pulse energy attenuation (factors of 3 and 10) to prevent excessive depletion of the precursor ions (saturation) and minimize the formation of fragment ions with mass below the low-mass cutoff of the quadrupole ion trap, which would result in underestimated IRMPD intensities.^20^

Trial molecular structures were optimized by using the Molecular Mechanics (MM2) option in Chem3D software. The optimized structures for each unique conformer of RF monomer, dimer, and photoproducts, with the lowest calculated energy along with one other distinctive geometric structure for comparison were selected for optimization at the DFT level of theory (B3LYP/6-311G­(d,p)) in Gaussian 16.^14^ All reported structures correspond to true minima, as confirmed by the frequency calculations. The B3LYP functional was chosen as it has been used successfully in a similar IRMPD study of protonated riboflavin.^15^ Its ability to predict accurate vibrational frequencies makes it particularly suitable for calculating the IR spectra of such systems. However, we note that the B3LYP functional can result in increased errors of the calculated energies, particularly in larger molecules.^24–26^

Matching of spectra against a computed spectrum is necessary for unbiased structure elucidation as well as the classification of spectra. A direct method of matching is to compare, point by point, two digitized spectra, the outcome being a parameter that quantifies the degree of similarity or dissimilarity between the spectra.^27^ All calculations conducted here were compared to the experimental spectra using Pearsons’ correlation coefficient (PCC), which measures the linear correlation between two sets of data. It gives the ratio between the covariance of two variables and the product of their standard deviations, providing a normalized measurement of the covariance and a result that has a value between −1 and 1.^28^

The Pearson correlation coefficient is defined as,

2 $$\rho \left(A,B\right)=\frac{1}{N-1}\sum Ni=1\left(\frac{A_{i}-\mu_{A}}{\sigma_{A}}\right)\left(\frac{B_{i}-\mu_{B}}{\sigma_{B}}\right)$$

where *μ*_*A*_ and *σ*_*A*_ are the mean and standard deviation of *A*, respectively, and *μ*_*B*_ and *σ*_*B*_ are the mean and standard deviation of *B*. The PCC is used here to compare calculated peaks to the experimentally measured spectra for riboflavin and its photoproducts. For each comparison, the spectra can be shifted by ±100 cm^–1^ in order to optimize the result of the PCC fit and remain within the range of a reasonable shift for an IR spectra.

## 3 Results

### 3.1 Electrospray Ionization of Riboflavin

A full ion mass spectrum obtained upon electrospray ionization of a solution of riboflavin is shown in Figure 1. Intense peaks are visible at *m*/*z* 375, 255, and 751, which can be assigned to deprotonated riboflavin [RF-H]^−^, lumiflavin [LF-H]^−^, and the riboflavin dimer [RF-H]*^–^* ·RF, respectively. The *m*/*z* 255 ion, [LF-H]^−^, is formed as the sole product of collision-induced dissociation of [RF-H]^−^ (Figure S1), so its appearance can be attributed to in-source dissociation:

3 $${[\mathrm{RF‐H}]}^{‐}\left(m/z\;375\right)\to {[\mathrm{LF‐H}]}^{‐}\left(m/z\;255\right){+C}_{4}H_{8}O_{4}$$

**Figure 1:**
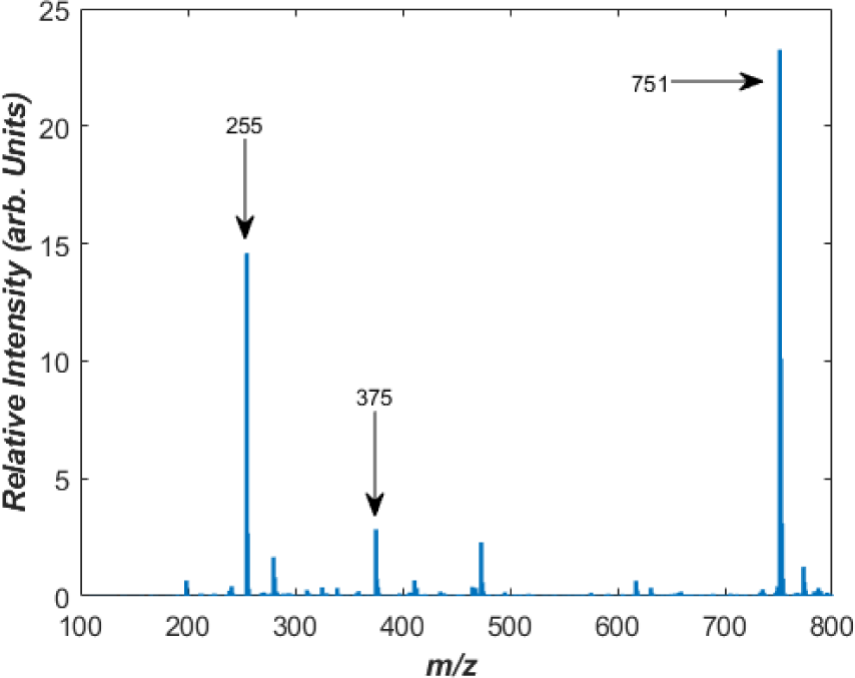
Full ion mass spectrum of riboflavin in HPLC-grade (H_2_O), with a 2 μL drop of (NH_3_) to aid deprotonation. Spectrum is recorded in negative ion mode without exposure to UV radiation. Peaks for [RF*-*H]^−^ (*m*/*z* 375), [LF*-*H]^−^ (*m*/*z* 255), and [RF*-*H]^−^·RF (*m*/*z* 751) are labeled.

In addition to the riboflavin dimer complex, [RF-H]^−^·RF (*m*/*z* 751), we also readily observed trimer clusters, i.e., [RF-H]^−^·RF_2_, albeit at lower intensity than that of the dimer cluster (Figure S2). The intensity of dimer complex, and other aggregates, is a sensitive function of pH. Section S2 provides further details.

### 3.2 Time Resolved Mass Spectrometry of Solution-Phase Photolysis of Riboflavin

Figure 2 displays the relative ion intensities observed via online ESI-MS when riboflavin in solution is photolyzed at 365 nm, displayed as a function of the photolysis time. To record this data, the electrospray syringe was tightly covered in light-tight foil for 2 min, and the foil was then removed simultaneously with the UV photodiode being turned on (current 0.7 A). The relative ion intensities that are initially present upon electrospray (*m*/*z* 255, 375, and 751) remained constant for 5 min, consistent with a 3 min transit time of the photolyzed solution into the mass spectrometer. Photoproducts and intermediates of the photodegradation reaction are then readily identifiable due to the variation in their intensities.

**Figure 2:**
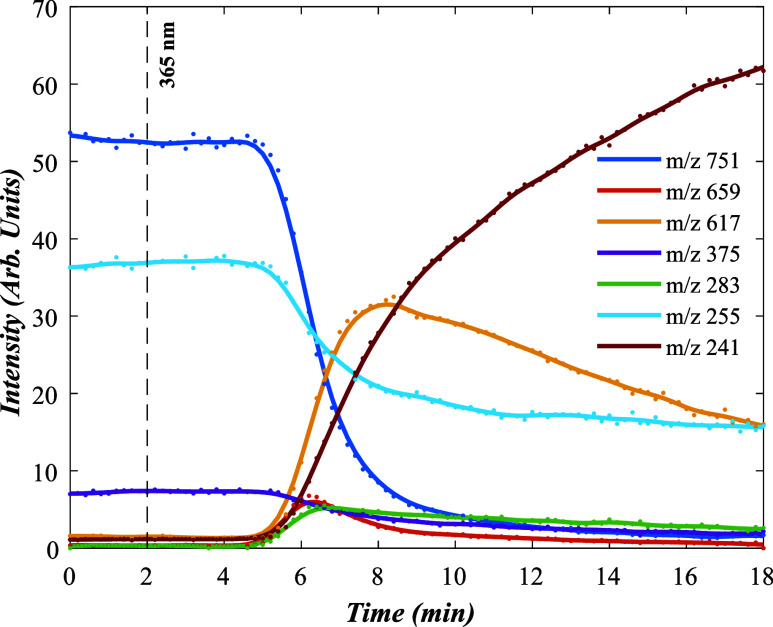
ESI-MS ion intensities observed for 365 nm photolysis of solution-phase riboflavin are displayed as a function of time. Photolysis is initiated at *t* = 2 min and there is a 3 min lead time for the solution to reach the MS.

After 5 min, the intensities of the initial precursor ions, *m*/*z* 375, 255, and 751, can be seen to begin decreasing, which continues over the period between *t* = 5 and 7 min. Concomitant with the fall in intensities of these precursor ions, the intensity of the major photofragment with *m*/*z* 241, which is assigned as deprotonated lumichrome, [LC-H]*^–^*, is seen to grow steadily, with its intensity starting to plateau only beyond *t* > 19 min. Eq 4 depicts the production of [LC-H]*^–^* from a riboflavin precursor:

$${[\mathrm{RF‐H}]}^{‐\;}\left(m/z\;375\right)+h\nu \to {[\mathrm{LC‐H}]}^{‐\;}\left(m/z\;241\right){+C}_{5}H_{10}O_{4}$$ 4

Three other ions can be seen to change in intensity across the time window studied, namely, *m*/*z* 659, 617, and 283, with all three of these ions increasing in intensity after *t* = 5 min, before then decreasing in intensity at differential rates, marking them as intermediates in the full photolysis reaction. The *m*/*z* 283 ion can be assigned as the deprotonated form of formylmethylflavin (FMF), i.e., [FMF-H]^−^, which has been previously identified as a product in aqueous reaction pathways of riboflavin.^29–31^ Eq 5 depicts its formation as a photolysis intermediate:

5 $${[\mathrm{RF‐H}]}^{‐}\;\left(m/z\;375\right)+h\nu \to {[\mathrm{FMF‐H}]}^{‐\;}\left(m/z\;283\right){+C}_{3}H_{8}O_{3}$$

The *m*/*z* 617 photofragment can be assigned as a deprotonated dimer of riboflavin and lumichrome, [RF-H]^−^·LC, formed via:

6 $${[\mathrm{RF‐H}]}^{‐}\cdot \mathrm{RF}\left(m/z\;751\right)+h\nu \to {[\mathrm{RF‐H}]}^{‐}\cdot \mathrm{LC}\left(m/z\;617\right){+C}_{5}H_{10}O_{4}$$

with the *m*/*z* 659 intermediate similarly being a deprotonated dimer of riboflavin and FMF, i.e., [RF-H]^−^·FMF:

7 $${[\mathrm{RF‐H}]}^{‐}\cdot \mathrm{RF}\left(m/z\;751\right)+h\nu \to {[\mathrm{RF‐H}]}^{‐}\cdot \mathrm{FMF}\left(m/z\;659\right){+C}_{3}H_{8}O_{3}$$

It is notable from the time-resolved profiles that [RF-H]^−^·LC (*m*/*z* 617) appears earlier and with a higher intensity than [RF-H]^−^·FMF (*m*/*z* 659). This is consistent with either two separate photolysis pathways for [RF-H]^−^·RF producing the two distinct photoproducts, i.e., both ([Disp-formula eq6]) and ([Disp-formula eq7]), or with some of the [RF-H]^−^·FMF being an intermediate for production of further [RF-H]^−^·LC.

Intriguingly, the time-resolved profiles show that the [RF-H]^−^·LC (*m*/*z* 617) moiety begins to form at a time before the [RF-H]^−^ (*m*/*z* 751) monomer photolyzes, and [RF-H]^−^·LC (*m*/*z* 617) is produced earlier than the [LC-H]^−^ (*m*/*z* 241), indicating that dimer photolysis is enhanced compared to that of the monomer. Similarly, [RF-H]^−^·FMF (*m*/*z* 659) forms slightly before and decays at a faster rate than [FMF-H]^−^ (*m*/*z* 283). These observations indicate that photolysis of the dimer is enhanced compared to the monomer, with the respective *t*_1/2_ values being 6.4 and 8.6 min.

While the riboflavin monomer and dimer display different photolysis rates, they demonstrate the same behavior in relation to the photoproducts they produce: [RF-H]^−^ can decay either directly to [LC-H]^−^ or via [FMF-H]^−^ to either [LC-H]^−^ or [LF-H]^−^, while [RF-H]^−^·RF decays either directly to [LC-H]^−^·RF or via [FMF-H]^−^·RF. Therefore, the intrinsic excited-state decay pathways of riboflavin remain similar for the monomer and dimer. Previous photochemical studies of riboflavin have shown that formylmethylflavin, lumichrome, and lumiflavin are formed from triplet-state excitation, whereas the first singlet excited state directly forms LC.^29,32^ Given that we observe both direct LC formation and indirect formation via FMF, it appears that the 365 nm excitation employed here accesses both the singlet and the triplet excited states.

### 3.3 IR Spectra of [RF-H]^−^, [RF-H]^−^·RF, and Their Photoproducts

IRMPD spectra of [RF-H]^−^, [RF-H]^−^·RF, and their main photoproducts are presented in Figure 3, to allow for a comparison of the spectra for the various species. All of the spectra display peaks in the region 1450–1600 cm*^–^* ^1^, associated with C–N stretches of heterocyclic rings, along with a group of intense peaks between 1650–1750 cm*^–^* ^1^ associated with C=O stretches. Some of the spectra also display peaks in the region below 1400 cm^–1^, where C–O^–^ hydroxide stretches are expected to occur. (We were unable to acquire the IRMPD of the *m*/*z* 659 ion, as its intensity was lower and it was more transitory than the other species studied.)

**Figure 3:**
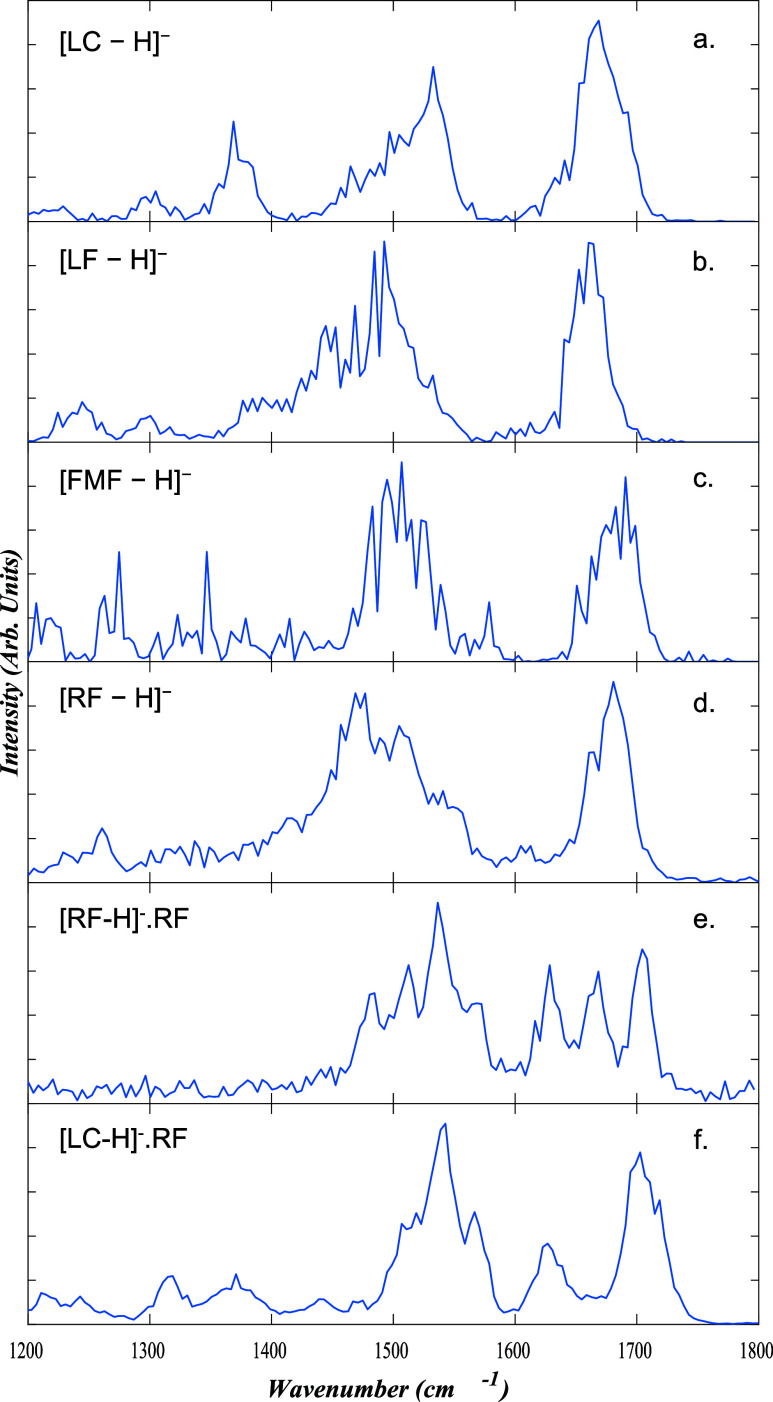
IRMPD spectra across the range 1200 to 1800 cm^–1^ for a) [LC-H]^−^, b) [LF-H]^−^, c) [FMF-H]^−^, d) [RF-H]^−^, e) [RF-H]^−^·RF, and f) [LC-H]^−^·RF.

#### 3.3.1 [LC*-*H]^−^

Lumichrome is the simplest member of the flavin family and represents the core chromophore. It is also the main riboflavin photoproduct (Section 3.2). Figure 4 presents the IRMPD spectrum of [LC-H]^−^ (*m*/*z* 241), obtained by IR-induced fragmentation into a single *m*/*z* 198 fragment ion, corresponding to the loss of HNCO from the uracil ring.^21,33^

**Figure 4:**
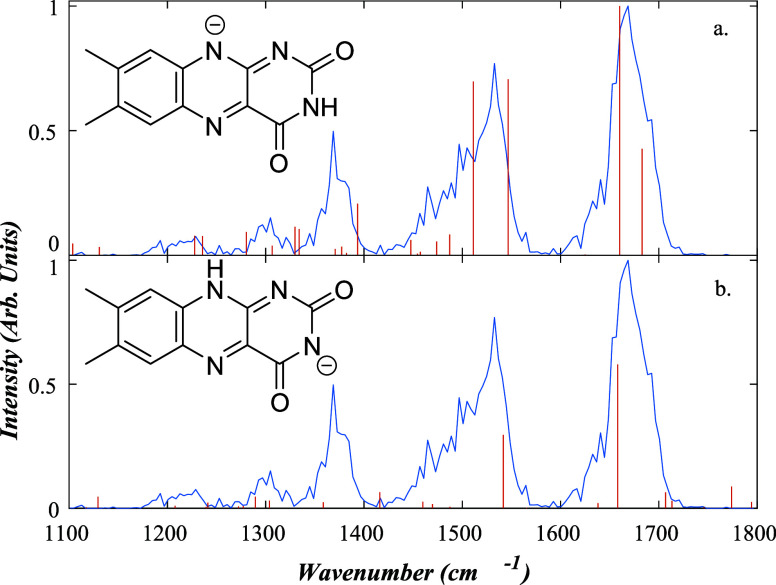
Measured IRMPD spectrum for [LC-H]^−^ (blue) shown with the calculated spectra (red) for the a) [LC-H_N10_]^−^ and b) [LC-H_N3_]^−^ deprotomers. For a) PCC = 0.77; shift = −64.08 cm^*–*1^; relative zero-point energy 2.74 kJ/mol: For b) PCC = 0.44; shift = 6.32 cm^*–*1^; relative zero-point energy 0 kJ/mol.

[LC*-*H]^−^ exhibits two primary deprotonation sites at positions N10 and N3 (Scheme 1). N1 can also be viewed as a deprotonation site, but N1 deprotonation is equivalent to N10 since the excess charge delocalizes over these two positions if either N carries an excess negative charge. (Excess charge is also partially delocalized, in this case, onto the two oxygen atoms for the N3 isomer.) Previous calculations at the PBE0/6-311+G­(d,p) level have shown that the N10/N1 deprotomer is the lower-energy isomer compared to N3 in both the gas phase and in solution.^21^ Calculations conducted in the current work predict that the N3 isomer has the lowest relative energy, with the N10 isomer lying 2.74 kJ/mol higher in energy. (It has been demonstrated in a prior study that the relative energies of deprotomers are very sensitive to the exact geometric structures, and the discrepancy in relative energies in calculations using B3LYP and PBE0 is consistent with this.^34^)

The structures for the N10 and N3 [LC*-*H]^−^ deprotomers and their associated calculated IR spectra are shown in Figure 4, along with the experimental spectrum. Five main peaks are visible in the experimental spectrum at 1668, 1532, 1368, 1304, and 1228 cm*^–^* ^1^. The calculated spectrum for [LC*-*H_N10_]^−^ has a PCC value of 0.77, indicating a very good fit. Comparing the [LC*-*H_N10_]^−^ experimental and calculated spectra, the calculated vibrations that match the five main peaks of the experimental spectrum are as follows: 1676 cm*^–^* ^1^ (C=O stretch), 1525 cm*^–^* ^1^ (C=N stretch), 1372 cm*^–^* ^1^ (C*–*N stretch), 1305 cm*^–^* ^1^ (N*–*H bend), and 1200 cm*^–^* ^1^ (C–N stretch), along with a very low-intensity peak at 1601 cm*^–^* ^1^ (C=C stretch). In comparison, the fit of the [LC*-*H_N3_]^−^ deprotomer has a PCC value of 0.44, with the agreement between the calculated and experimental spectrum being poor across the 1400–1600 cm*^–^* ^1^ region, due to the perturbed structure of the uracil ring following N3 deprotonation. This led us to conclude that [LC*-*H_N10_]^−^ is the dominant deprotomer in the experimental ion ensemble.

Since the [LC-H]^−^ moiety is a key structural unit for the subsequent systems analyzed below, it is useful to summarize the IRMPD features associated with it, namely broad, intense peaks at ∼1650 and 1550 cm^–1^, due to two vibrations occurring in each of these regions, along with a lower intensity but still prominent peak at ∼1380 cm^–1^.

#### 3.3.2 [LF*-*H]^−^

Figure 5 presents the IRMPD spectrum of deprotonated lumiflavin [LF*-*H]^−^ (*m*/*z* 254.76) obtained following fragmentation in the *m*/*z* 239.72, 211.76, and 196.66 fragment ions. The highest-intensity fragment is *m*/*z* 239.72, followed by *m*/*z* 196.66, with *m*/*z* 211.76 appearing with only very low intensity.

**Figure 5:**
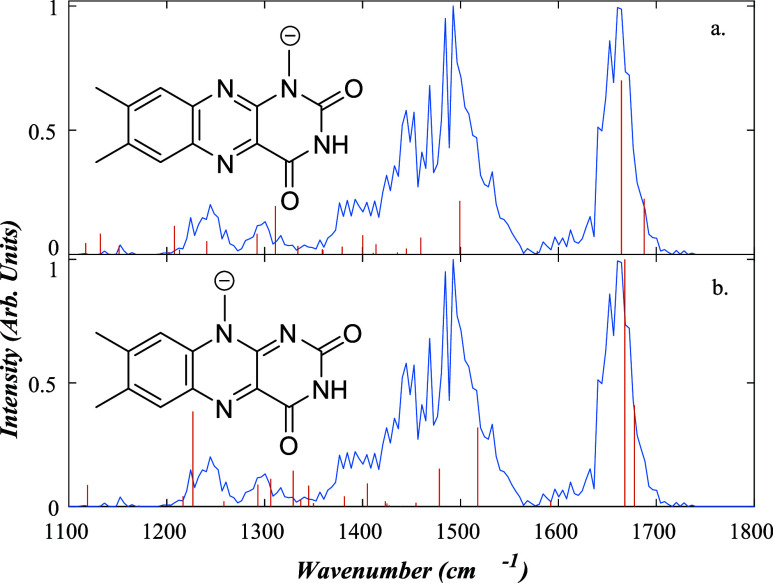
Measured IRMPD spectrum for [LF-H]^−^ (blue) shown with the calculated spectra (red) for a) [LF*-*H_Me‑N1_]^−^ and b) [LF*-*H_Me‑N10_]^−^. For a) PCC = 0.48; shift = −55.09 cm*^–^* ^1^; relative zero-point energy 0 kJ/mol: For b) PCC = 0.34; shift = −66.07 cm*^–^* ^1^; relative zero-point energy 5.42 kJ/mol.

Lumiflavin has two primary deprotonation sites associated with either the N3 site or the methyl group attached to the N10 position. [LF-H]^−^ can also exist as an alloxazine or isoalloxazine isomer dependent on the R group being on N1 or N10, respectively. From our [LC*-*H]^−^ results, deprotonation is unlikely to take place at N3, so we will not consider that deprotomer further here. Our calculations predict that [LF-H_Me‑N1_]^−^ and [LF-H_Me‑N10_]^−^ have relative energies of 0 and 5.42 kJ/mol, respectively. Calculated IR spectra are presented for the two isomers in Figure 5, along with the experimental spectrum. It is evident that the calculated spectra for the two isomers are similar, making it challenging to assign the spectrum unambiguously to a single isomer. However, it is clear that the experimental spectrum is not consistent with N3 deprotonation, since such a structure is associated with very low vibrational intensity below 1400 cm^–1^ (Figure S4). Considering the PCC spectral matches, the [LF-H_Me‑N1_]^−^ and [LF-H_Me‑N10_]^−^ isomers have values of 0.48 and 0.34, respectively. These values are rather low, due mainly to the poor replication of the intensity of the calculated spectral bands. However, given that the PCC value is higher for [LF-H_Me‑N1_]^−^, we tentatively assigned the IRMPD spectrum to this alloxazine isomer.

The [LF*-*H]^−^ IRMPD spectrum has major peaks at 1660, 1532, 1492–1468, 1412–1372, 1300, and 1244 cm*^–^* ^1^. For [LF*-*H_Me‑N10_]^−^, the calculated vibrations at 1664 and 1687 cm*^–^* ^1^ correspond to an asymmetric C=O stretch and an N–H bend on the uracil group. Peaks at 1517 and 1478 cm*^–^* ^1^ represent the C=N and C=C stretches of the alloxazine and the peaks from 1404 to 1226 cm*^–^* ^1^ correspond to C–C and C–N stretches with C–H bends on the alloxazine ring. For [LF*-*H_Me‑N1_]^−^, similar vibrations are calculated to appear at 1600 and 1678 cm*^–^* ^1^, with additional vibrations corresponding to C=C and C=N stretches at 1499 and 1459 cm*^–^* ^1^, and C*–*N bends and stretches at 1413 to 1240 cm*^–^* ^1^.

#### 3.3.3 [FMF-H]^−^

Deprotonated formylmethylflavin, [FMF-H]^−^ (*m*/*z* 282.91), is a photochemical intermediate of riboflavin (Section 3.2), produced by the loss of C_3_H_8_O_3_. FMF has two primary deprotonation sites: the N_3_ site, common to all flavins, or the alkyl hydrogen atoms of the carbon side chain. Since [LC-H]^−^ and [LF-H]^−^ do not show a propensity for deprotonation at the N_3_ site, it is reasonable to expect that the same will be true for [FMF-H]^−^ and that deprotonation will therefore occur on the side chain. Our calculated relative energies support this argument, with [FMF*-*H_C1_]^−^ and [FMF*-*H_N3_]^−^ having relative energies of 0 and 4.92 kJ/mol. While we do not expect to observe the N3 deprotomer, we have shown the calculated spectrum for this isomer in Figure 6, along with the one for the side-chain deprotomer to provide a comparison.

**Figure 6:**
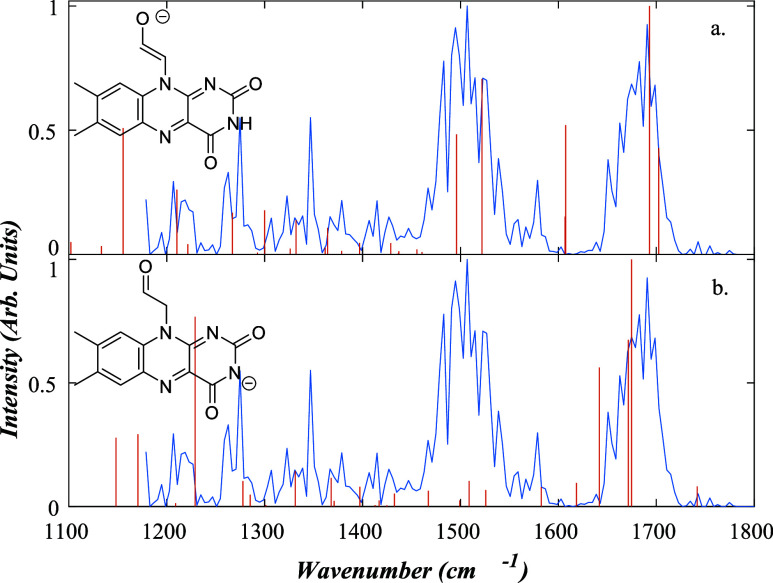
Measured IRMPD spectrum for [FMF-H]^−^ (blue) shown with the calculated spectra (red) for a) the [FMF-H_C1_]^−^, and b) the [FMF-H_N3_]^−^ deprotomers. For a) PCC = 0.45, shift = −56.16 cm*^–^* ^1^, and relative zero-point energy 0 kJ/mol, while for b) PCC = 0.25, shift = 5.84 cm*^–^* ^1^, relative zero-point energy 4.92 kJ/mol.

It was challenging to obtain the IRMPD spectrum for [FMF-H]^−^ due to it being a photochemical intermediate that was only present at certain time points. Indeed, it had the lowest ion intensity of the ions subjected to IR spectroscopic interrogation in this study, and the experimental spectrum displayed in Figure 6 therefore has a lower signal-to-noise ratio than the other spectra presented in this work.

The [FMF*-*H_C1_]^−^ calculated structure provides the best match to the experimental spectrum with a PCC of 0.45, compared to that for [FMF*-*H_N3_]^−^ of 0.25. However, the PCC fit for [FMF*-*H_C1_]^−^ is still relatively poor, leading us to consider the spectral match further. The relatively broad experimental feature located at 1690 cm*^–^* ^1^ corresponds to the excitation of the two C=O stretches on the uracil group. Both sets of calculations predict peaks in this region: [FMF*-*H_C1_]^−^ has peaks at 1702 and 1693 cm*^–^* ^1^, while [FMF*-*H_N3_]^−^ has peaks at 1674, 1671, and 1641 cm*^–^* ^1^ (all asymmetric C=O stretches). In addition, [FMF*-*H_N3_]^−^ has an additional lower-intensity peak at 1741 cm*^–^* ^1^ associated with a symmetric stretch of the uracil carbonyls. Over the region around 1690 cm^–1^, these factors lead to the [FMF*-*H_N3_]^−^ deprotomer, providing a better fit to experiment. However, this is not the case over other spectral regions. This isomer should display a characteristic C=N=O bend on the uracil ring at 1228 cm*^–^* ^1^, which again is not evident in the experimental spectrum. These particular factors do not occur when comparing the [FMF*-*H_C1_]^−^ fit to experiment, and in addition, the lone peak observed at 1578 cm*^–^* ^1^ on the experimental spectrum can be assigned to a shift in the double peak at 1607 cm*^–^* ^1^ in the [FMF*-*H_C1_]^−^ calculation, which gives a C=O stretch and a C*–*H bend on the R-group. This leads us to conclude that the spectrum can be largely attributed to the [FMF*-*H_C1_]^−^ deprotomer, due to both the better spectral match and its lower zero-point energy.

#### 3.3.4 [RF-H]^−^

Figure 7a presents the IRMPD spectrum for [RF-H]^−^ (*m*/*z* 375), which is acquired via IRMPD of [RF-H]^−^ into the *m*/*z* 255, 241, and 212 fragments, corresponding to [LF-H]^−^, [LC-H]^−^, and [AL-H]^−^, respectively.

**Figure 7:**
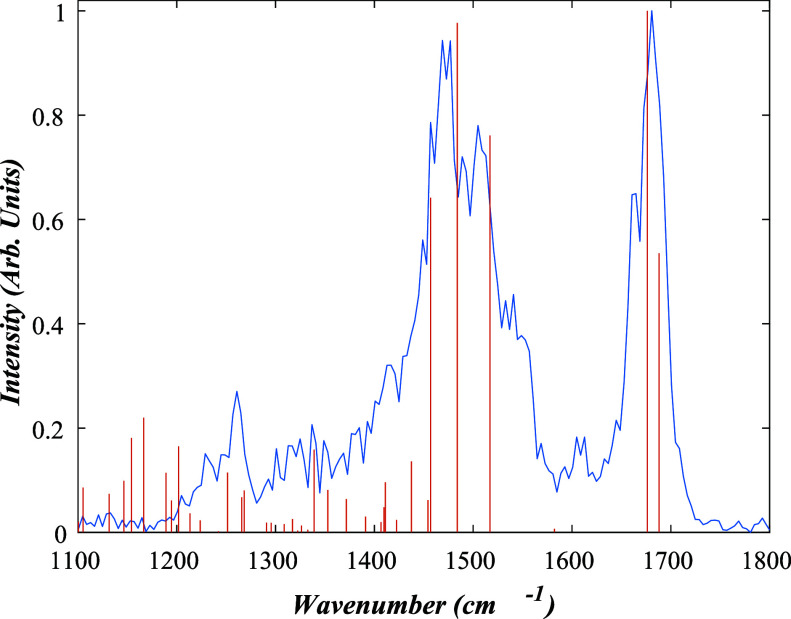
Measured IRMPD spectrum for [RF*-*H]^−^ (blue) is shown with the calculated spectra (red) for the [RF*-*H_OH1_]^−^ deprotomer. For PCC = 0.66; shift = −85.48 cm*^–^* ^1^; relative zero-point energy 0 kJ/mol.

It is interesting to first compare the [RF-H]^−^ spectrum to that of [LC-H]^−^ (Figure 3). The spectra are broadly similar, with each showing broad peaks around 1700 and 1570 cm^–1^, but with the [LC-H]^−^ showing increased vibrational activity in the spectral region just below 1500 cm^–1^. The absence of a vibration in the region of 1400 cm^–1^ is consistent with the functionalization of the N10 position, in contrast to the nonderivatized [LC-H]^−^. Of the structures we calculated, the highest PCC fit value (0.66) was obtained for the [RF*-*H_OH1_]^−^ isomer, which displayed the lowest relative energy. To provide a comparison, Table S4 presents the spectral fit data for this isomer along with the one for the N3 deprotomer which has a significantly lower PCC value of 0.43, and deprotonation at the remaining OH groups of the ribityl chain. The better fit for the [RF*-*H_OH1_]^−^ deprotomer leads us to assign the experimental system to this isomer.

The ribityl chain of [RF-H]^−^ leads to distinctive IR vibrations for this ion compared to the smaller systems discussed above, with the calculated O*–*H bends of the sugar chain appearing in the 1300–1450 cm*^–^* ^1^ region for both deprotomers, albeit at relatively low intensity. The double-peak feature between 1630 and 1700 cm*^–^* ^1^ is present in the simulated spectrum for [RF*-*H_OH1_]^−^, where these features correspond to two individual C=O stretches on the uracil of the alloxazine ring.

The simulated and experimental spectra over the 1400–1600 cm*^–^* ^1^ region match reasonably well for both deprotomers, although the agreement is clearly better for [RF*-*H_OH1_]^−^. This spectral region peaks correspond to the C–H bends and C=C stretches of the aromatic ring system of the alloxazine. The peaks in the 1300–1500 cm*^–^* ^1^ region also correspond to the O–H vibrations on the ribityl chain.

#### 3.3.5 [RF-H]^−^·RF

The *m*/*z* 751 ion corresponds to the singly deprotonated riboflavin dimer, which we anticipate corresponds to an [RF-H]^−^ anion that has been deprotonated on the ribityl chain, noncovalently bound to a neutral RF. It is likely that the ribityl chains of the two RF molecules will hydrogen bond to one another, in a manner that allows intermolecular dispersion interactions to occur between the two alloxazine rings.

Figure 8 displays the IRMPD spectrum obtained for [RF-H]^−^·RF, showing two intense groups of vibrations between 1470 and 1580 cm^–1^ and 1600–1750 cm^–1^. We anticipate that the two intense groups of vibrations will similarly be associated with ribityl OH vibrations and aromatic rings and C=O stretches for [RF-H]^−^·RF. It is notable that both types of vibration have blue-shifted upon the complexation of [RF-H]^−^ to RF. The 1630–1750 cm^–1^ region corresponds to C=O stretches of the uracil group. We see three peaks here due to the presence of the second alloxazine ring combined with intermolecular interactions such as hydrogen bonding or pi-stacking of the alloxazine rings. Comparing the spectra, there are lower intensities relative to [RF-H]^−^ and spectral red-shifts for some peaks. The 1470–1580 cm^–1^ peaks are more distinct than the peaks of the [RF-H]^−^ spectrum due to the increased number of O–H bends on the two ribityl chains combined with red-shifting due to the intermolecular and intramolecular hydrogen bonding.

**Figure 8:**
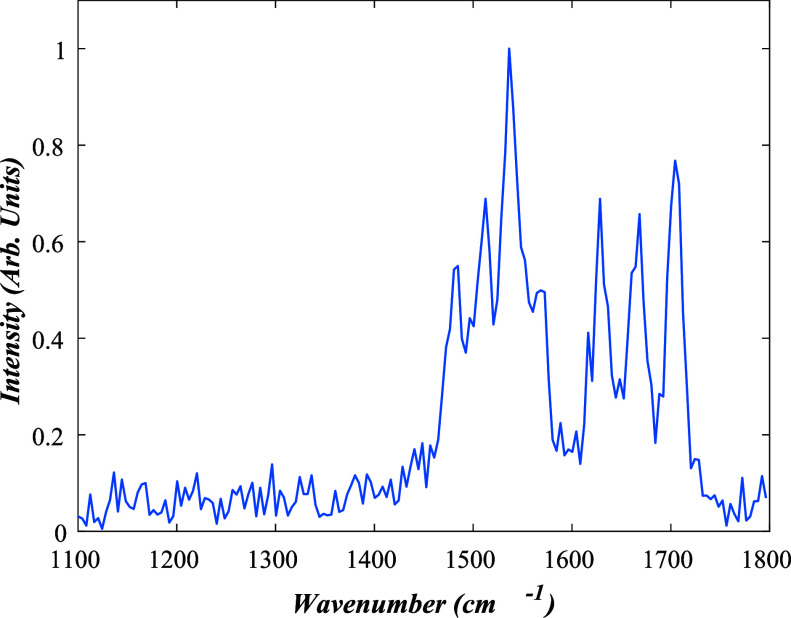
IRMPD spectrum of the *m*/*z* 751 ion assigned to [RF-H]^−^·RF.

Figure S5 shows the measured [RF-H]^−^·RF, compared to the calculated combined spectra for [RF*-*H_OH1_]^−^ and a separate, neutral RF molecule. In this simulated spectrum, three distinct peaks are visible in both the 1600–1700 and 1470–1570 cm^–1^ regions, indicating that a combined aggregate of neutral and deprotonated riboflavin molecules gives a spectral fingerprint that is consistent with the observed experimental spectrum.

#### 3.3.6 *m*/*z* 617: [RF·LC-H]^−^

The photochemical intermediate with *m*/*z* 617 has been assigned above as a deprotonated dimer of RF and LC. This is likely produced when a riboflavin dimer photodegrades with one of the RF molecules decaying to LC via ejection of the ribityl chain, but the resulting [RF·LC-H]^−^ complex is only metastable as the second RF molecule can subsequently absorb a photon and photodegrade into a further lumichrome with accompanying cluster photodissociation.

Using the same arguments as presented above for the dimer [RF-H]^−^·RF, we assume that [RF·LC-H]^−^ will be deprotonated either on the RF ribityl side chain or at the N10 position of the lumichrome. Since [LC-H]^−^ is formed by the loss of neutral ribityl from RF, it seems likely that this cluster corresponds to [LC-H]^−^, although it is possible that the negative charge moves from the LC to the RF even if deprotonated LC is initially formed photochemically.

Figure 9 presents the IRMPD spectrum of the *m*/*z* 617 ion. Comparing the spectrum to that of [LC-H]^−^, it is notable that both spectra share similar spectral features in the regions of 1700 and 1550 cm^–1^. There is also considerable similarity between the two spectra in the region below 1400 cm^–1^. One of the main differences between the two spectra is the appearance of a peak at 1550 cm^–1^ in the [RF·LC-H]^−^ spectrum. This feature was not seen in either the [LC-H]^−^ spectrum or the [RF-H]^−^. It is possible that this vibration is associated with an OH mode of the neutral ribityl side chain that would be present in an [LC-H]^−^ cluster. It is also notable that the two most intense peaks in the [RF·LC-H]^−^ spectrum at 1550 and 1700 cm^–1^ are again blue-shifted compared to the analogous peaks in the [LC-H]^−^ spectrum.

**Figure 9:**
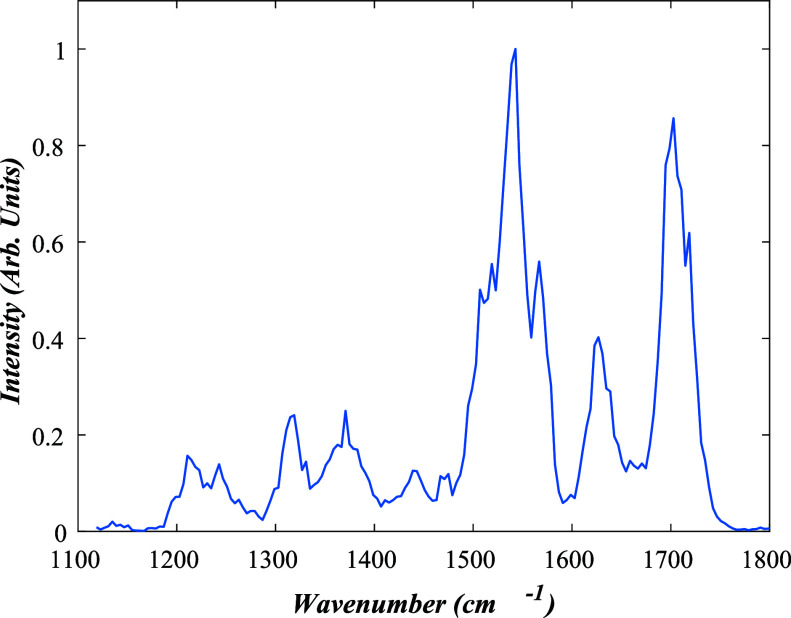
IRMPD spectrum of the *m*/*z* 617 ion assigned as [LC-H]^−^·RF.

The main features of the experimental spectrum show the usual groups of the aromatic system and OH bends at 1506–1566 cm*^–^* ^1^ and the C=O stretches on the uracil group in the 1700 cm*^–^* ^1^ range. However, the peak in the 1622 cm*^–^* ^1^ region has a higher intensity than those in any of the other experimentally recorded flavin IRMPD spectra. The calculations also show a higher intensity peak at 1630 cm*^–^* ^1^, which is the N*–*H bend at the N10 position on the lumichrome. As none of the other flavins presented have this group, it is reasonable to assume that this structure for the riboflavin-lumichrome dimer is correct. A comparison of the combined calculated spectra of a neutral RF with [LC-H_N10_]^−^ is shown in Figure S6, along with a combined calculated spectrum for a neutral LC molecule with [RF*-*H_OH1_]^−^ (Figure S7). These simulated spectra clearly show that the experimental spectrum is more consistent with an assignment of the *m*/*z* 617 ion as [LC-H]^−^·RF, as anticipated.

## 4 Further Discussion

IRMPD over the fingerprint region has been employed here to spectroscopically identify the key photoproducts (lumichrome and lumiflavin) and the photochemical intermediate (formylmethylflavin) observed following 365 nm photolysis of riboflavin. Furthermore, IRMPD allowed us to characterize the composition of the singly deprotonated riboflavin dimer, [RF-H]^−^·RF, and the photochemical intermediate *m*/*z* 617 ion as RF·[LC-H]^−^. The identification of *m*/*z* 617, along with the structure of the [RF-H]^−^·RF dimer itself is important as it shows that the photochemical pathway followed by the riboflavin dimer following photoexcitation mirrors that of the riboflavin monomer, i.e., chromophore excitation of an RF unit, followed by excited-state decay into lumichrome with ejection of the neutral ribityl chain.

The IRMPD spectra recorded in this study provide clear assignments of the structures of the deprotomers present following ESI. However, the electrospray process can produce ratios of protomeric and deprotomeric isomers that differ from those present in the solution, introducing some uncertainty in the assumption that the gas-phase assignment aligns with the solution-phase composition. As a general rule, when ESI happens with a nonprotic solvent, the solution-phase population of protomers/deprotomers is preserved, whereas when a protic solvent is employed, proton exchange can occur during ESI and the protomers/deprotomers observed will correspond to those that are the lowest-energy gas-phase isomers.^33,35–37^ The relative energies of the N10 and N3 deprotomers of LC have calculated previously for both the solution phase and gas phase,^21^ showing that the N10 deprotomer is the lower-energy isomer in both the solution and gas phases. We can therefore be confident that the deprotomers we observed here both [LC-H]^−^ and RF·[LC-H]^−^ are present in solution, as well as under the gas-phase IRMPD spectroscopy conditions. This may well also be the case for the other flavins studied here since deprotonation on the N3 position is likely to remain unfavored in both the solution and gas phases. Nonetheless, the role of the ESI processes in influencing the isomers present in the gas phase is an important one to consider in studies such as this.

This investigation provides the first direct evidence of the role played by the riboflavin dimer in accelerating photochemical degradation through the observation of the deprotonated dimer decaying more rapidly following photoexcitation at 365 nm than the deprotonated monomer.

The effect of noncovalent interactions on the properties of flavins is well documented,^38–45^ and these interactions are key to the ability of flavins to bind to proteins and modulate their redox properties.^43,46,47^ Although flavins are known to display a propensity to aggregate,^48^ to our knowledge, this is the first direct observation of how aggregation affects riboflavin’s photochemical properties. The accelerated photodegradation of riboflavin that we observe here upon aggregation could potentially arise from spectral shifting of the electronic transition(s) in the vicinity of 365 nm, leading to an enhanced absorption coefficient for the dimer compared to the monomer.^49^ Gas-phase electronic spectroscopy of RF·[RH-H]^−^ along with quantum chemical calculations would be useful to confirm this hypothesis and provide a more nuanced picture of how the first singlet and first triplet states are perturbed upon aggregation. More generally, it is interesting to reflect on the fact that the accelerated photodegradation we observe here with aggregation almost certainly is a key factor in the light-induced degradation of riboflavin-containing foodstuffs.^50^ Tailored strategies for minimizing flavin aggregation have been employed in photocatalysis work with flavin analogues,^51^ and the fundamental insight gained in the current study may be valuable in further refining such efforts.

## Supplementary Material



## Notes

Collision Induced Dissociation of [RF-H]^−^; ESI-MS of RF and dependence on pH; Calculated IR spectrum for the N3 deprotomer of [LF-H]^−^; Calculated IR spectrum of neutral RF; Simulated IR spectra for a selection of flavin complexes; Computational chemistry results for deprotonated LC, LF, FMF and RF (PDF)

## References

[1] Conrad K. S., Manahan C. C., Crane B. R. (2014). Photochemistry of Flavoprotein Light
Sensors. Nat. Chem. Biol..

[2] Knak A., Regensburger J., Maisch T., Bäumler W. (2014). Exposure of
Vitamins to UVB and UVA Radiation Generates Singlet Oxygen. Photochem. Photobiol. Sci..

[3] Srivastava V., Singh K., Srivastava P., Singh A. P. (2021). Synthetic Applications
of Flavin Photocatalysis: A Review. RSC Adv..

[4] Wong N. G.
K., Rhodes C., Dessent C. E. H. (2021). Photodegradation of Riboflavin under
Alkaline Conditions: What Can Gas-Phase Photolysis Tell Us about What
Happens in Solution?. Molecules.

[5] Peechakara, B. V. ; Sina, R. E. ; Gupta, M. Vitamin B2 (Riboflavin). In StatPearls; StatPearls Publishing: 2024. 30247852

[6] O’Callaghan B., Bosch A. M., Houlden H. (2019). An Update on the Genetics,
Clinical
Presentation, and Pathomechanisms of Human Riboflavin Transporter
Deficiency. J. Inherited Metab. Dis..

[7] Insińska-Rak M., Golczak A., Sikorski M. (2012). Photochemistry of Riboflavin Derivatives
in Methanolic Solutions. J. Phys. Chem. A.

[8] Insińska-Rak M., Prukała D., Golczak A., Fornal E., Sikorski M. (2020). Riboflavin
Degradation Products; Combined Photochemical and Mass Spectrometry
Approach. J. Photochem. Photobiol., A.

[9] Bairagi A., Pereverzev A. Y., Tinnemans P., Pidko E. A., Roithová J. (2024). Electrocatalytic
CO2 Reduction: Monitoring of Catalytically Active, Downgraded, and
Upgraded Cobalt Complexes. J. Am. Chem. Soc..

[10] Edington S. C., Perez E. H., Charboneau D. J., Menges F. S., Hazari N., Johnson M. A. (2021). Chemical Reduction of NiII Cyclam and Characterization
of Isolated NiI Cyclam with Cryogenic Vibrational Spectroscopy and
Inert-Gas-Mediated High-Resolution Mass Spectrometry. J. Phys. Chem. A.

[11] Moons P. H., Ter Braak F., de Kleijne F. F. J., Bijleveld B., Corver S. J. R., Houthuijs K. J., Almizori H. R., Berden G., Martens J., Oomens J. (2024). Characterization of
Elusive Rhamnosyl Dioxanium Ions and Their Application in Complex
Oligosaccharide Synthesis. Nat. Commun..

[12] Tripodi G. L., Derks M. T. G. M., Rutjes F. P. J. T., Roithová J. (2021). Tracking Reaction
Pathways by a Modular Flow Reactor Coupled to Electrospray Ionization
Mass Spectrometry. Chem.: Methods.

[13] Thomas G. T., Donnecke S., Chagunda I. C., McIndoe J. S. (2022). Pressurized Sample
Infusion. Chem.: Methods.

[14] Langer J., Günther A., Seidenbecher S., Berden G., Oomens J., Dopfer O. (2014). Probing Protonation
Sites of Isolated Flavins Using
IR Spectroscopy: From Lumichrome to the Cofactor Flavin Mononucleotide. ChemPhyschem.

[15] Müller D., Dopfer O. (2021). Interaction of Alkali
Ions with Flavins: Infrared and
Optical Spectra of Metal–Riboflavin Complexes. J. Phys. Chem. A.

[16] Nieto P., Günther A., Berden G., Oomens J., Dopfer O. (2016). IRMPD Spectroscopy
of Metalated Flavins: Structure and Bonding of Lumiflavin Complexes
with Alkali and Coinage Metal Ions. J. Phys.
Chem. A.

[17] Giacomozzi L., Kjær C., Brøndsted Nielsen S., Ashworth E. K., Bull J. N., Stockett M. H. (2021). Non-Statistical
Fragmentation in
Photo-Activated Flavin Mononucleotide Anions. J. Chem. Phys..

[18] Matthews E., Cercola R., Dessent C. E. H. (2018). Protomer-Dependent Electronic Spectroscopy
and Photochemistry of the Model Flavin Chromophore Alloxazine. Molecules.

[19] Bull J. N., Carrascosa E., Giacomozzi L., Bieske E. &., Stockett M. H. (2018). Ion Mobility
Action Spectroscopy of Flavin Dianions Reveals Deprotomer-Dependent
Photochemistry. Phys. Chem. Chem. Phys..

[20] Berden G., Derksen M., Houthuijs K. J., Martens J., Oomens J. (2019). An Automatic
Variable Laser Attenuator for IRMPD Spectroscopy and Analysis of Power-Dependence
in Fragmentation Spectra. Int. J. Mass Spectrom..

[21] Matthews E., Dessent C. E. H. (2018). Observation of
Near-Threshold Resonances in the Flavin
Chromophore Anions Alloxazine and Lumichrome. J. Phys. Chem. Lett..

[22] Uleanya K. O., Anstöter C. S., Dessent C. E. H. (2023). Photodissociative Decay Pathways
of the Flavin Mononucleotide Anion and Its Complexes with Tryptophan
and Glutamic Acid. Phys. Chem. Chem. Phys..

[23] Martens J., Berden G., Gebhardt C. R., Oomens J. (2016). Infrared Ion Spectroscopy
in a Modified Quadrupole Ion Trap Mass Spectrometer at the FELIX Free
Electron Laser Laboratory. Rev. Sci. Instrum..

[24] Shao Y., Mei Y., Sundholm D., Kaila V. R. I. (2020). Benchmarking the Performance of Time-Dependent
Density Functional Theory Methods on Biochromophores. J. Chem. Theory Comput..

[25] Chen L., Süß D., Sukuba I., Schauperl M., Probst M., Maihom T., Kaiser A. (2020). Performance of DFT
Functionals for Properties of Small Molecules Containing Beryllium,
Tungsten and Hydrogen. Nucl. Mater. Energy.

[26] Altürk S., Avcı D., Tamer Ö, Atalay Y. (2017). Comparison of Different
Hybrid DFT Methods on Structural, Spectroscopic, Electronic and NLO
Parameters for a Potential NLO Material. Comput.
Theor. Chem..

[27] Li J., Hibbert D. B., Fuller S., Vaughn G. (2006). A Comparative Study
of Point-to-Point Algorithms for Matching Spectra. Chemom. Intell. Lab. Syst..

[28] Samuel A. Z., Mukojima R., Horii S., Ando M., Egashira S., Nakashima T., Iwatsuki M., Takeyama H. (2021). On Selecting a Suitable
Spectral Matching Method for Automated Analytical Applications of
Raman Spectroscopy. ACS Omega.

[29] Sheraz M. A., Kazi S. H., Ahmed S., Anwar Z., Ahmad I. (2014). Photo, Thermal
and Chemical Degradation of Riboflavin. Beilstein
J. Org. Chem..

[30] Smith E. C., Metzler D. E. (1963). The Photochemical
Degradation of Riboflavin. J. Am. Chem. Soc..

[31] Ahmad I., Fasihullah Q., Vaid F. H. M. (2006). Photolysis of Formylmethylflavin
in Aqueous and Organic Solvents. Photochem.
Photobiol. Sci..

[32] Ahmad I., Fasihullah Q., Noor A., Ansari I. A., Ali Q. N. M. (2004). Photolysis
of Riboflavin in Aqueous Solution: A Kinetic Study. Int. J. Pharm..

[33] Steill J. D., Oomens J. (2009). Gas-Phase Deprotonation
of p-Hydroxybenzoic Acid Investigated
by IR Spectroscopy: Solution-Phase Structure Is Retained upon ESI. J. Am. Chem. Soc..

[34] Wong N. G. K., Rankine C. D., Dessent C. E. H. (2021). Measurement
of the Population of
Electrosprayed Deprotomers of Coumaric Acids Using UV–Vis Laser
Photodissociation Spectroscopy. J. Phys. Chem.
A.

[35] Matthews E., Dessent C. E. H. (2017). Experiment and Theory Confirm That
UV Laser Photodissociation
Spectroscopy Can Distinguish Protomers Formed via Electrospray. Phys. Chem. Chem. Phys..

[36] Matthews E., Dessent C. E. H. (2016). Locating the
Proton in Nicotinamide Protomers via Low-Resolution
UV Action Spectroscopy of Electrosprayed Solutions. J. Phys. Chem. A.

[37] Schröder D., Buděšínský M., Roithová J. (2012). Deprotonation
of P-Hydroxybenzoic Acid: Does Electrospray Ionization Sample Solution
or Gas-Phase Structures?. J. Am. Chem. Soc..

[38] McDonald N.
A., Subramani C., Caldwell S. T., Zainalabdeen N. Y., Cooke G., Rotello V. M. (2011). Simultaneous
Hydrogen Bonding and
π-Stacking Interactions between Flavin/Porphyrin Host–Guest
Systems. Tetrahedron Lett..

[39] Caldwell S. T., Cooke G., Hewage S. G., Mabruk S., Rabani G., Rotello V., Smith B. O., Subramani C., Woisel P. (2008). Model Systems for Flavoenzyme Activity:
Intramolecular
Self-Assembly of a Flavin Derivative Via hydrogen Bonding and Aromatic
Interactions. Chem. Commun..

[40] Ju S.-Y., Papadimitrakopoulos F. (2008). Synthesis
and Redox Behavior of Flavin Mononucleotide-Functionalized
Single-Walled Carbon Nanotubes. J. Am. Chem.
Soc..

[41] Butterfield S. M., Goodman C. M., Rotello V. M., Waters M. L. (2004). A Peptide Flavoprotein
Mimic: Flavin Recognition and Redox Potential Modulation in Water
by a Designed β Hairpin. Angew. Chem.,
Int. Ed..

[42] Gray M., Goodman A. J., Carroll J. B., Bardon K., Markey M., Cooke G., Rotello V. M. (2004). Model Systems for Flavoenzyme Activity:
Interplay of Hydrogen Bonding and Aromatic Stacking in Cofactor Redox
Modulation. Org. Lett..

[43] Pellett J. D., Becker D. F., Saenger A. K., Fuchs J. A., Stankovich M. T. (2001). Role of
Aromatic Stacking Interactions in the Modulation of the Two-Electron
Reduction Potentials of Flavin and Substrate/Product in *Megasphaera
Elsdenii* Short-Chain Acyl-Coenzyme A Dehydrogenase. Biochemistry.

[44] Staab H. A., Kanellakopulos J., Kirsch P., Krieger C. (1995). Π···π
Interactions of Flavins, 5. Syntheses, Structures and Physical Properties
of Flavin Systems with Covalent Bonding to π Donors and π
Acceptors (Quinones). Liebigs Ann..

[45] Niemz A., Rotello V. M. (1999). From Enzyme to Molecular Device. Exploring the Interdependence
of Redox and Molecular Recognition. Acc. Chem.
Res..

[46] Collard F., Fagan R. L., Zhang J., Nemet I., Palfey B. A., Monnier V. M. (2011). The Cation−π Interaction between Lys53
and the Flavin of Fructosamine Oxidase (FAOX-II) Is Critical for Activity. Biochemistry.

[47] Estarellas C., Frontera A., Quiñonero D., Deyà P. M. (2011). Anion-π
Interactions in Flavoproteins. Chem. - Asian
J..

[48] Drabent R., Grajek H. (1983). The Flavin Dimers I.
The Application of Absorption
in Anti-Stokes Excitation Region to Investigate the Flavin Dimer Formation. Biochim. Biophys. Acta, Gen. Subj..

[49] Astanov S. K., Kasimova G. K., Kurtaliev E. N., Nizomov N. N., Jumabaev A. (2021). Electronic
Nature and Structure of Aggregates of Riboflavin Molecules. Spectrochim. Acta, Part A.

[50] Choe E., Huang R., Min D. B. (2005). Chemical Reactions
and Stability
of Riboflavin in Foods. J. Food Sci..

[51] Dad′ová J., Kümmel S., Feldmeier C., Cibulková J., Pažout R., Maixner J., Gschwind R. M., König B., Cibulka R. (2013). Aggregation Effects in Visible-Light Flavin Photocatalysts:
Synthesis, Structure, and Catalytic Activity of 10-Arylflavins. Chem. - Asian J..

