# Supplementary material for: Spectroscopic Characterization of the Photolysis of Riboflavin (Vitamin B2) via Time-Resolved Mass Spectrometry and IRMPD Spectroscopy
Source: J Phys Chem A. 2025 May 29;129(23):5082–91. doi: 10.1021/acs.jpca.5c02175 (PMC12169675; doi:10.1021/acs.jpca.5c02175)
Supplement: Supplementary file 1 [file jp5c02175_si_001.pdf]

Supporting Information for Publication

Spectroscopic Characterization of the Photolysis of Riboflavin (Vitamin B2) via Time-Resolved Mass Spectrometry and IRMPD Spectroscopy

Sarah A. Wilson,[*,†] Aljawharah Alsalem,[†] Geil Berden,[‡] Jos Oomens,[‡] and Caroline E.H. Dessent[*,†]

†Department of Chemistry, University of York, York, United Kingdom

‡ High Field Magnet Laboratory (HFML - EMFL), Radboud University, Toernooiveld 7, 6525 ED Nijmegen, The Netherlands.

*Corresponding authors : E-mail: sarah.wilson3@york.ac.uk; caroline.dessent@york.ac.uk

Contents

S1. Collision Induced Dissociation of $[RF-H]^-$

S2. ESI-MS of RF and dependence on pH

S3. Calculated IR spectrum for the N3 deprotomer of $[LF-H]^-$

S4: Calculated IR spectrum of neutral RF

S5: Simulated IR spectra for a selection of flavin complexes

S6: Computational chemistry results for deprotonated LC, LF, FMF and RF

S1. Collision Induced Dissociation of $[RF-H]^-$

Collision-induced dissociation (CID) of the $[RF-H]^-$ precursor ion (*m/z* 375) dissociates solely into $[LF-H]^-$ (*m/z* 255) as shown in Figure S1. The precursor $[RF-H]^-$ does not dissociate up to 10% CID, indicating that the ion is relatively stable against spontaneous dissociation, and its only fragment is $[LF-H]^-$.

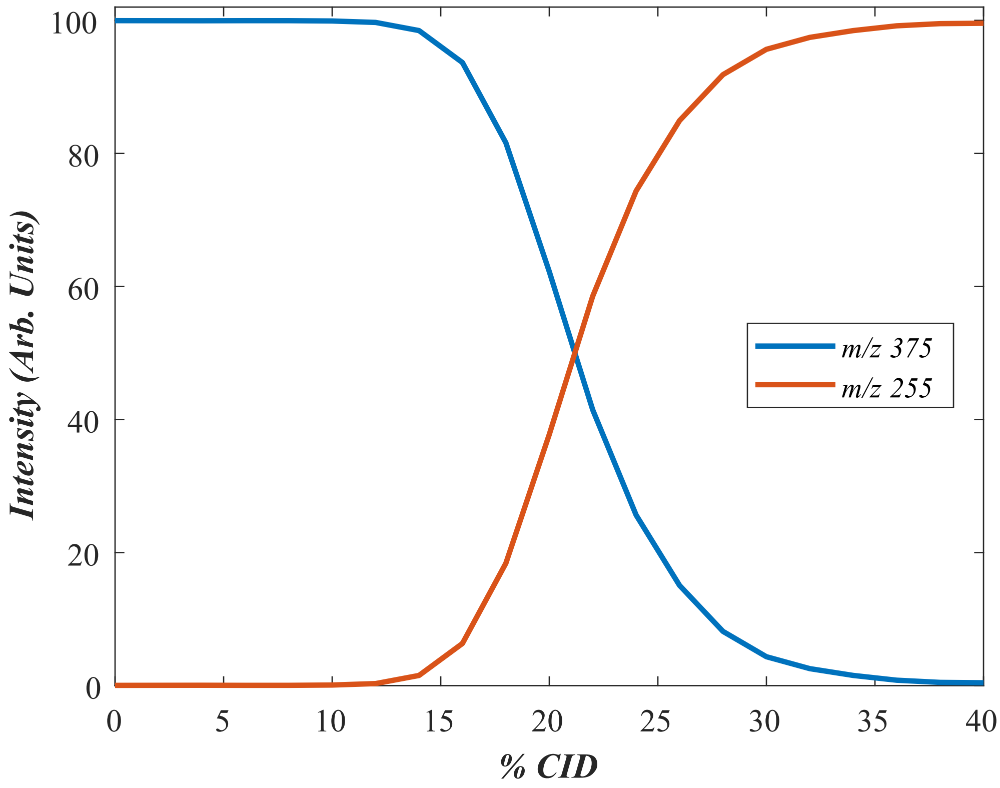

Figure S1: Collision-induced dissociation (CID) fragmentation decay curve for $[RF-H]^-$ (blue) showing fragments of *m/z* 255 (red), upon CID between 0% and 40% CID energy.

S2. ESI-MS of RF and dependence on pH

A full ion spectrum of a solution of Riboflavin (RF) in HPLC-grade (H_2O), with a 2μL drop of (NH3) to aid deprotonation, in negative ion mode is shown in Figure S2. The spectrum has peaks at *m/z* 375, 255, 751 and 1126. These correspond to deprotonated riboflavin $[RF\text{-}H]^-$, lumiflavin $[LF\text{-}H]^-$, the riboflavin dimer $[RF\text{-}H]^-{\cdot}RF$ and the riboflavin trimer $[RF\text{-}H]^-{\cdot}(RF)_2$, respectively.

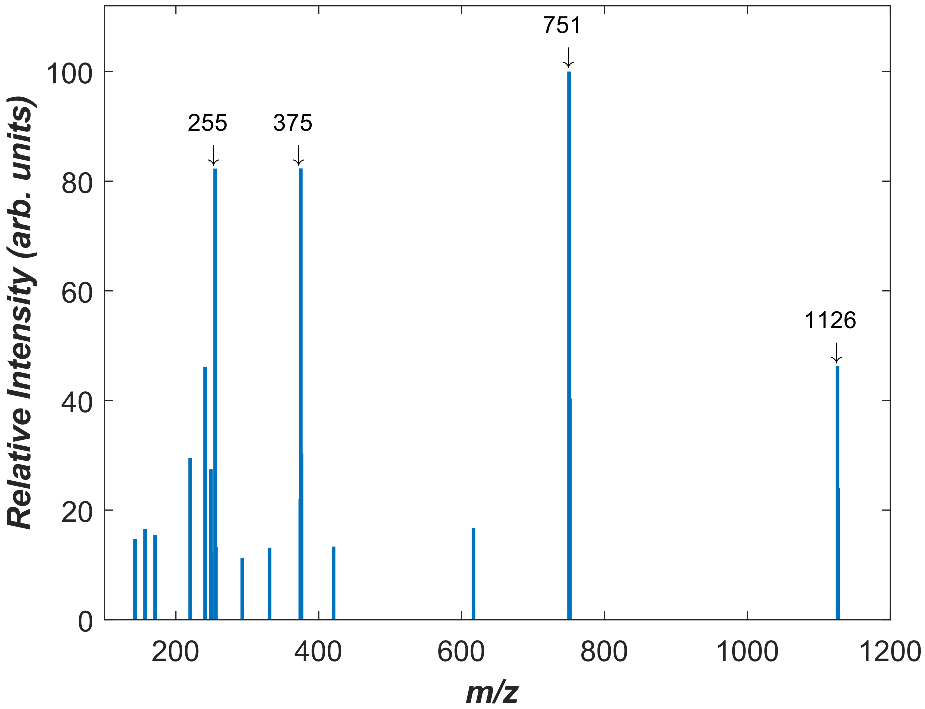

Figure S2: Full ion mass spectrum of Riboflavin without exposure to UV radiation. Peaks for $[RF-H]^-$ (*m/z* 375), $[LF-H]^-$ (*m/z* 255), $[RF-H]^-{\cdot}RF$ (*m/z* 751), $[RF-H]^-{\cdot}(RF)_2$ (*m/z* 1126) are labelled.

To explore how production of the dimer complex varies with pH of the precursor solution, we obtained electrospray ionisation mass spectra (ESI-MS) of riboflavin at a variety of pH by varying the amount of ammonia solution added to the riboflavin solution. Figure S3 shows how the relative intensity of $[RF\text{-}H]^-{\cdot}RF$ varies relative to the deprotonated riboflavin monomer, $[RF\text{-}H]^-$, illustrating that the dimer/monomer ratio decreases as the amount of ammonia (and hence pH) increases. As the pH increases, the propensity for both RF moieties of the RF dimer to be deprotonated increases, but a doubly deprotonated dimer, *i.e.* $[RF\text{-}H]^{2-}$, would display a high propensity to dissociate, hence reducing the intensity of the dimer.

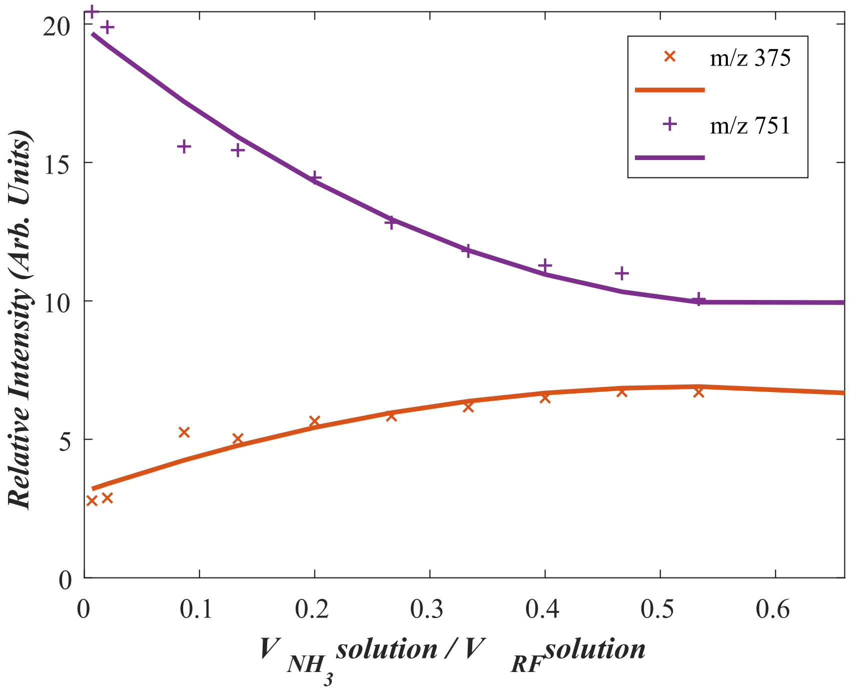

Figure S3: Relative intensities of [RF-H]$^-$ (*m/z* 375) and [RF-H]$^-$·RF (*m/z* 751) as a function of the volume of NH_3 solution in a 30 ml 10^{-6} mol/dm^3 solution of riboflavin in water.

Previous work by Wong *et. al.*[1] showed a very similar full ion spectra to Figure S2, however the Figure S2 spectrum shows a higher relative intensity of the *m/z* 751 dimer peak compared to the m/z 375 one. This can be understood from the data presented in Figure S3 as being due to a lower concentration of NH_3 solution having been used in the previous measurements.

Reference

1. Wong, N.G.K.; Rhodes, C.; Dessent, C.E.H. Photodegradation of Riboflavin under Alkaline Conditions: What Can Gas-Phase Photolysis Tell Us about What Happens in Solution? *Molecules* **2021**, *26*, 6009. https://doi.org/10.3390/molecules26196009

S3. Calculated IR spectrum for the N3 deprotomer of $[LF-H]^-$

Comparison of the experimental spectrum with the calculated spectrum for the N3 deprotomer of $[LF-H]^-$ (Figure S4) shows that the experimental spectrum cannot be attributed to this isomer, due to mismatch in spectral intensities below 1300 cm^{-1}. (The experimental spectrum has negligible intensity in this region, while the calculated spectrum predicts three moderate intensity vibrations between 1180-1260 cm^{-1}.)

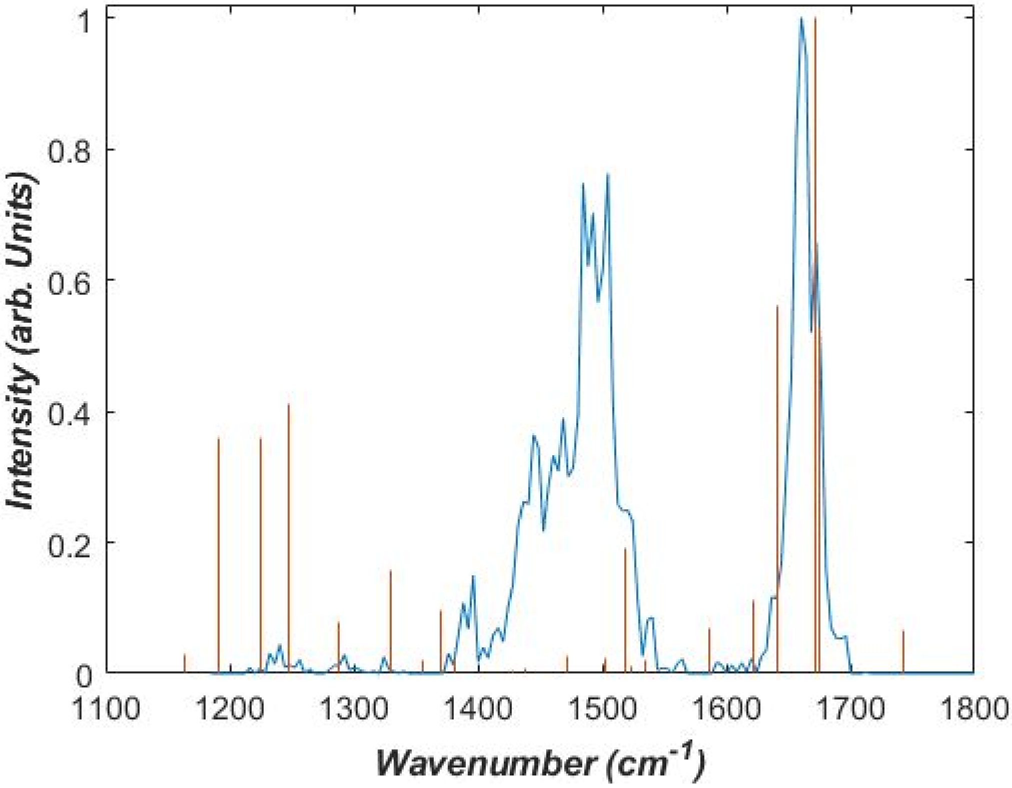

Figure S4: Measured IRMPD spectrum for $[LF-H]^-$ (blue) shown with the calculated IR spectrum (red) for $[LF-H_{N3}]^-$. PCC = 0.30; shift = 9.49 cm^{-1}; relative zero-point energy 4.63 kJ/mol:

S4. Calculated IR spectrum for neutral riboflavin

A geometric structure for neutral RF was generated following the same quantum chemical methods as used for the deprotonated ions detailed in the main text. The calculated IR spectrum for the neutral RF structure obtained is displayed in Figure S5.

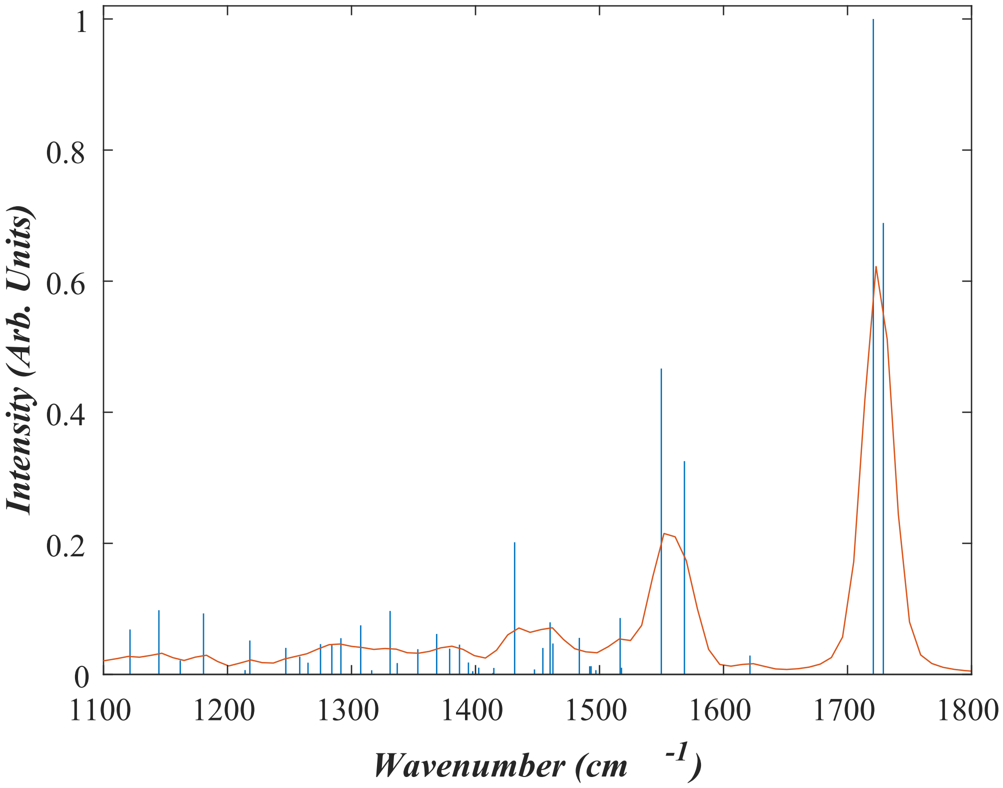

Figure S5: Calculated IR spectra of neutral Riboflavin.

S5. Simulated IR spectra for a variety of flavin complexes

S5.1 Experimental IRMPD spectrum and calculated IR spectrum for neutral riboflavin combined with the calculated spectrum for deprotonated [RF−H_{OH1}]$^-$

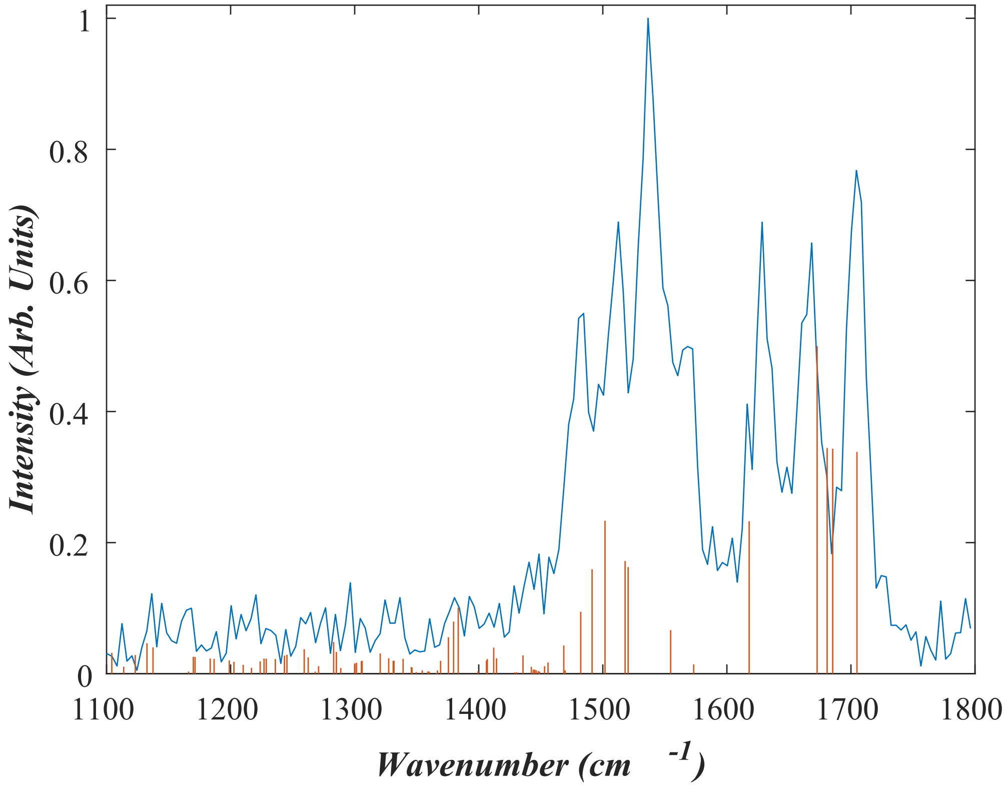

Figure S6: Measured IRMPD spectrum for [RF-H]$^-$·RF (blue) shown with the calculated spectra (red) for the [RF−H_{OH1}]$^-$ deprotomer and neutral RF (Figure S4) combined.

S5.2 Experimental IRMPD spectrum for [LC−H]⁻·RF shown with the calculated spectra for the combined [LC−H_{N10}]⁻ deprotomer and neutral RF

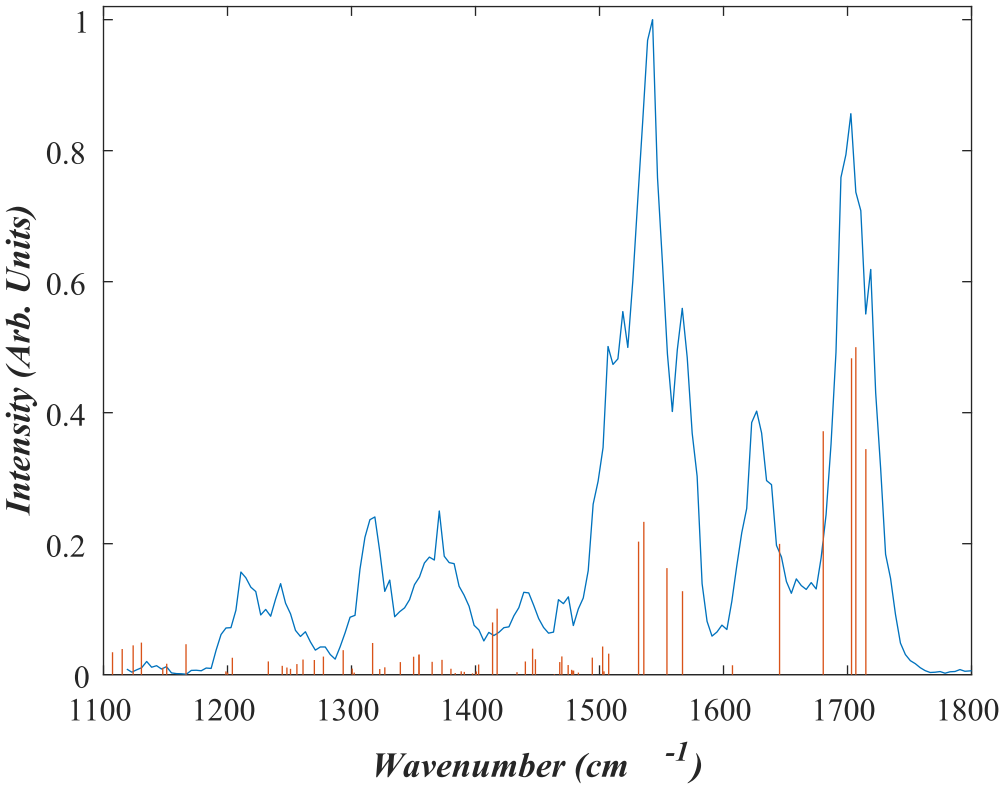

Figure S7: Measured IRMPD spectrum for [LC−H]⁻·RF (blue) shown with the calculated spectra (red) for the [LC-H_{N10}]⁻ deprotomer and neutral RF combined.

S4.3 Experimental IRMPD spectrum for [RF−H]⁻·LC and the calculated spectra for the combined [RF−H$_{OH1}$]⁻ deprotomer and neutral LC

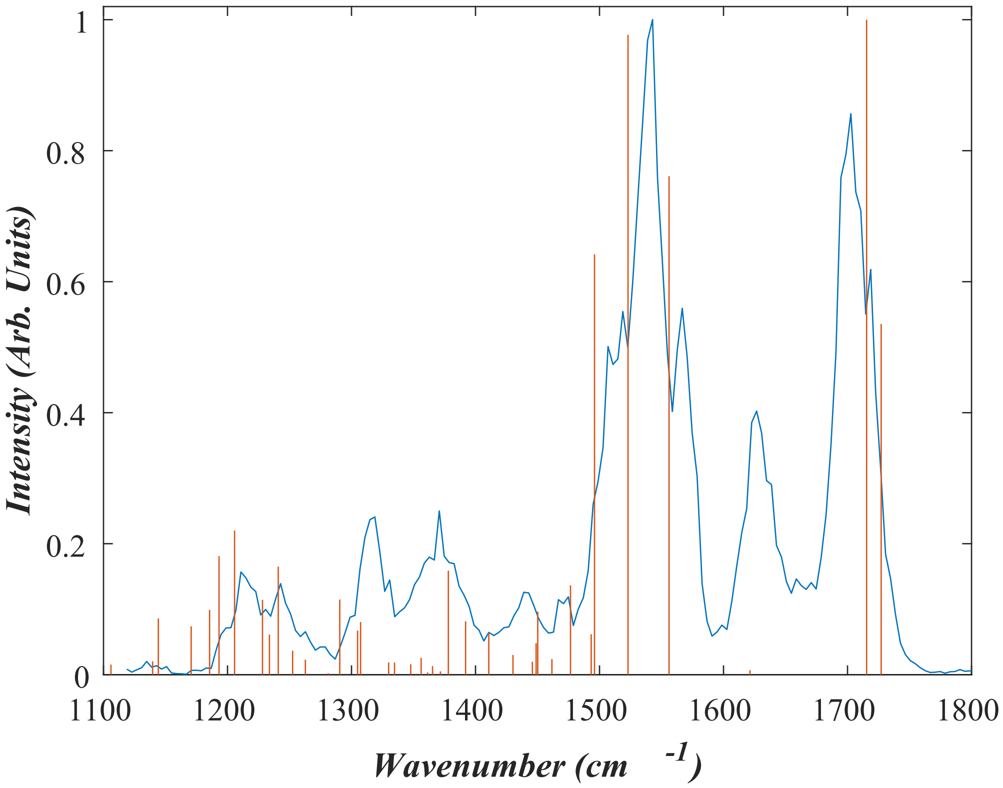

Figure S8: Measured IRMPD spectrum for [RF−H]⁻.LC (blue) shown with the calculated spectra (red) for the [RF−H$_{OH1}$]⁻ deprotomer and neutral LC combined.

S6. Computational chemistry results for deprotonated LC, LF, FMF and RF

The tables below give the computational chemistry results for the various isomers of deprotonated lumichrome, lumiflavin, FMF and riboflavin, obtained using the methods described in Section 2 of the main manuscript.

Table S1: Lumichrome

**Name**	**Structure**	**Deprotonation site**	**MM2 steric energy (kcal/mol)**	**Pearsons correlation coefficient**	**Relative zero point energy (kJ/mol)**
$[LC{-}H_{N10}]^-$	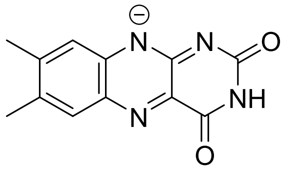	N10	7.84	0.76	2.74
$[LC{-}H_{N3}]^-$	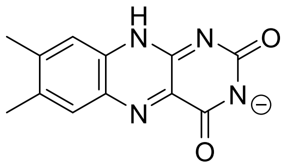	N3	61.44	0.44	0.00
$[LC{-}H_{N1}]^-$	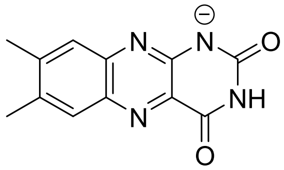	N1	9.79	0.76	2.74

Table S2: Lumiflavin

Name	Structure	Deprotonation site	MM2 steric Energy (kcal/mol)	Pearsons correlation coefficient	Relative zero point energy (kJ/mol)
$[LF{-}H_{N3\text{-}N10}]^{-}$	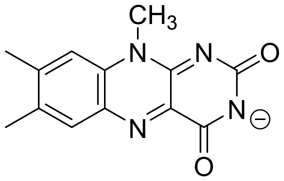	N3	71.60	0.33	4.63
$[LF{-}H_{Me\text{-}N10}]^{-}$	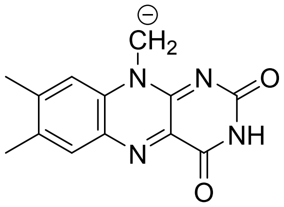	Me	24.45	0.34	5.42
$[LF{-}H_{N3\text{-}N1}]^{-}$	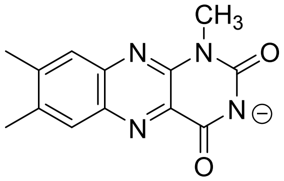	N3 (N1)	43.43	0.42	5.19
$[LF{-}H_{Me\text{-}N1}]^{-}$	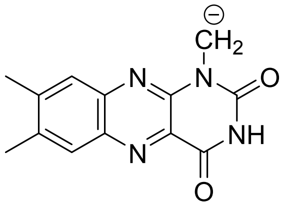	Me (N1)	26.12	0.46	0.00

Table S3: FMF

Name	Structure	Deprotonation site	MM2 steric energy (kcal/mol)	Pearsons correlation coefficient	Relative zero point energy (kJ/mol)
$[FMF-H_{C2}]^-$	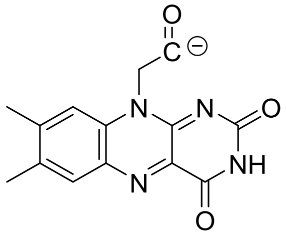	C1	23.05	0.45	4.92
$[FMF-H_{N3}]^-$	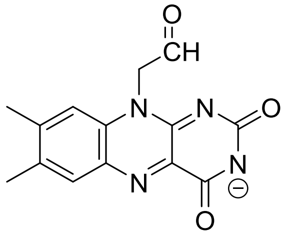	N3	74.03	0.59	0.00
$[FMF-H_{C1}]^-$	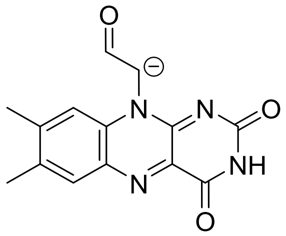	C2	22.98	0.45	11.04
$[FMF-H_{C1\text{-}N1}]^-$	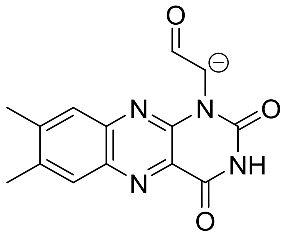	N1/C1	28.71	0.56	8.17
$[FMF-H_{C2\text{-}N1}]^-$	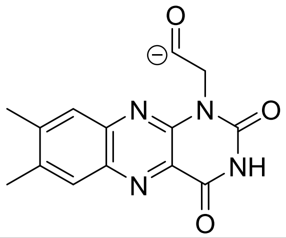	N1/C2	93.26	0.48	5.85

Table S4: Riboflavin

Name	Structure	Deprotonation site	MM2 steric energy (kcal/mol)	Pearsons correlation coefficient	Relative zero point energy (kJ/mol)
$[RF-H_{OH1}]^-$	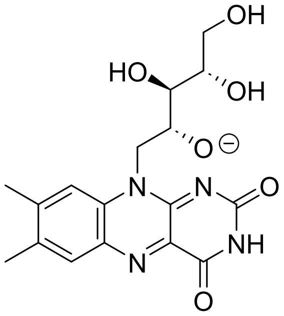	OH1	34.34	0.67	0.00
$[RF-H_{OH2}]^-$	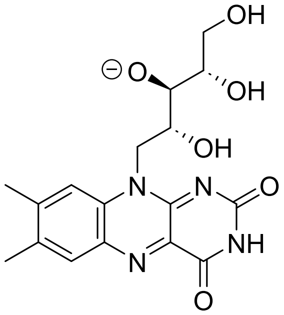	OH2	29.4	0.59	1.64
$[RF-H_{OH3}]^-$	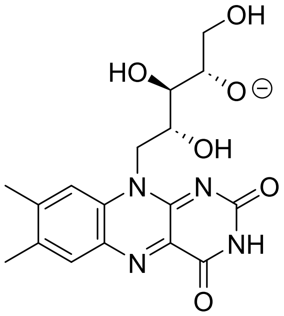	OH3	39.85	0.58	4.78
$[RF-H_{OH2}]^-$	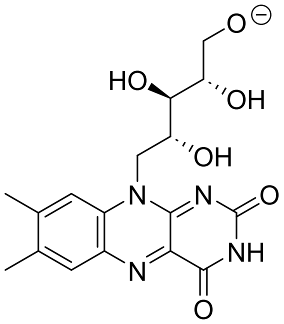	OH4	29.58	0.53	3.93
$[RF-H_{N3}]^-$	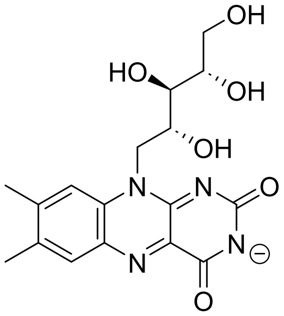	N3	75.29	0.43	4.36
